# Causes and characteristics of unexpected sudden cardiac death in octogenarians/nonagenarians

**DOI:** 10.1371/journal.pone.0284515

**Published:** 2023-04-20

**Authors:** Elisa Puolitaival, Juha Vähätalo, Lauri Holmström, M. Anette E. Haukilahti, Lasse Pakanen, Olavi H. Ukkola, M. Juhani Junttila, Heikki V. Huikuri, Juha S. Perkiömäki

**Affiliations:** 1 Research Unit of Biomedicine and Internal Medicine, Medical Research Center Oulu, University of Oulu and Oulu University Hospital, Oulu, Finland; 2 Finnish Institute for Health and Welfare, Forensic Medicine Unit, Oulu, Finland; 3 Department of Forensic Medicine, Research Unit of Biomedicine and Internal Medicine, Medical Research Center Oulu, University of Oulu, Oulu, Finland; BSMMU: Bangabandhu Sheikh Mujib Medical University, BANGLADESH

## Abstract

**Introduction:**

The risk for sudden cardiac death (SCD) increases with ageing.

**Methods:**

We evaluated causes and characteristics of unexpected SCD in SCD victims aged ≥ 80 years in a consecutive series of 5,869 SCD victims in Northern Finland. All the victims underwent medico-legal autopsy as medico-legal autopsy is mandatory in cases of unexpected sudden death in Finland. All the non-cardiac deaths such as pulmonary embolism and cerebral hemorrhage were excluded from the study, as were unnatural deaths such as intoxications.

**Results:**

Among SCD victims ≥ 80 years, 91.0% of SCDs were due to ischemic heart disease (IHD) determined in autopsy and 9.0% due to non-ischemic heart disease (NIHD), whereas among those < 80 years, only 72.6% of SCDs were due to IHD and 27.4% due to NIHD (P < .001). Severe fibrosis in myocardium was more common whereas heart weight and liver weight, body mass index and abdominal fat thickness, were lower among SCD victims aged ≥ 80 years than among victims aged < 80 years. In those with IHD as etiology of SCD, at least 75% stenosis in one or more major coronary vessels was more common in SCD victims aged ≥ 80 years than among victims aged < 80 years (P = .001). SCD victims 80 years or older were less likely to die during physical activity than those under 80 years old (5.6% vs. 15.9%, P < .001). Dying in sauna was more common among those ≥ 80 years than among those < 80 years (5.5% vs. 2.6%, P < .001).

**Conclusion:**

In victims of unexpected SCD aged ≥ 80 years, the autopsy-based etiology of SCD was more commonly IHD than in those aged < 80 years. In SCD victims aged ≥ 80 years, severe fibrosis in myocardium, representing arrhythmic substrate, was more common than in the younger ones.

## Introduction

It is well known that sudden cardiac death (SCD) is major cause of death in western societies. In 2017, 13.5% of all deaths in the United States were caused by SCD. Among males, SCD caused more deaths than other individual causes of deaths [1]. Up to half of the SCD victims have no prior diagnosis of cardiac disease before SCD [2]. Approximately 70–80% of SCDs are caused by coronary artery disease (CAD), although its contribution seems to have decreased in the last two decades. Cardiomyopathies are another important cause of SCD, and a minority of SCDs are due to inherited arrhythmic syndromes [2].

One of the major risk factors of SCD is aging. After adolescence, the risk of SCD increases exponentially with age. The mortality rates of SCD increase from 3.2/100,000 persons per year among 20- to 24-year-olds to 823.4/100,000 persons per year among those aged 80–84 years [1]. Even though the incidence of SCD has decreased in the last few decades, SCD will remain a major problem in healthcare as the population ages [2,3]. It is estimated that in 2030, 20% of USA population will be 65 years or older. Moreover, the fastest growth is expected to happen among 85 years and older [3].

Even though SCD is common in subjects ≥80 years old (octo-/nonagenarians), the data about the characteristics and causes of SCD in these subjects are limited. The present study was aimed to estimate the characteristics and causes of SCD in SCD victims aged 80 years or more. We compared the characteristic and causes of SCDs between SCD victims 80 years old or older and those under 80 years. We also compared the data between octogenarians (from 80 to 89 years) and nonagenarians (from 90 to 99 years) and between males and females aged ≥ 80 years.

## Methods

### Study population

The data was obtained from the large prospective Fingesture study (Finnish Genetic Study of Arrhythmic Events), which consists of a consecutive series of 5,869 victims of unexpected SCD in Northern Finland. The purpose of the study was to collect systematic information of all SCDs occurring in the study area. A medico-legal autopsy was performed on all victims by a forensic pathologist, as medico-legal autopsy is mandatory in cases of unexpected sudden death in Finland (Act on the Inquest into the Cause of Death, 459/973, 7^th^ paragraph: Finnish Law). The autopsies were performed between 1998–2017 at the Department of Forensic Medicine of the University of Oulu, Oulu, Finland and Finnish Institute for Health and Welfare, Oulu, Finland. In addition to post-mortem examination reports, the data about the SCD victims was collected from available medical records, police reports and standardized questionnaires filled by the closest family member of the victims.

The autopsies included both macroscopic and histologic examination. Macroscopic examination of heart included dissection of coronary arteries and myocardium, measuring heart weight and valve investigation. Biopsies for histological analysis were taken systemically from anterior, lateral and posterior wall of left ventricle, septum and right ventricle. A very experienced specialist in forensic medicine performed the autopsies and the classification of myocardial fibrosis in 4 stages in severity (substantial fibrosis, moderate patchy fibrosis, scattered mild fibrosis and no fibrosis) using standardized protocol. The determination of the degree of fibrosis was based on comprehensive evaluation of myocardial fibrosis in macroscopic myocardial dissection and histological tissue samples from several myocardial sites.

In this study, SCD was defined as witnessed death appearing within 6 hours of the onset of symptoms or death within 24 hours of last seen being alive if death was unwitnessed. All the non-cardiac deaths such as pulmonary embolism and cerebral hemorrhage were excluded from the study, as were unnatural deaths such as intoxications. Based on autopsy findings, the cause of SCD was classified as ischemic heart disease (IHD) or non-ischemic heart disease (NIHD). The cause of SCD, in victims with evidence of an active coronary artery process, defined as plaque rupture or erosion, acute intracoronary thrombus, intraplaque hemorrhage or coronary stenosis ≥ 75% in the major coronary artery or significant chronic atherosclerotic lesions with healed scar or fibrosis, was classified as IHD. The cause of SCD in cases that did not fulfill the criteria for IHD were classified as NIHD. The subcategories for nonischemic SCD deaths have been described in an earlier study by Haukilahti et al [4]. The methods of the Fingesture study are described in more detail in earlier studies [5,6].

The study complies with the declaration of Helsinki and was approved by the Ethics Committee of the University of Oulu and Finland’s Ministry of Social Affairs and Health. National Institute for Health and Welfare and National Supervisory Authority for Welfare and Health (Valvira) approved the investigators to review of autopsy data.

### Statistical analysis

All analyses were performed with the Statistical Package for Social Studies version 27. The statistical significance of differences between continuous variables was evaluated using the Independent Samples T Test. To assess the statistical significance of the differences between the categorical variables of the study groups, cross-tabulation and the chi-square test were used. All the P-values are two-sided and P < 0.05 was considered statistically significant.

## Results

**Fig 1:**
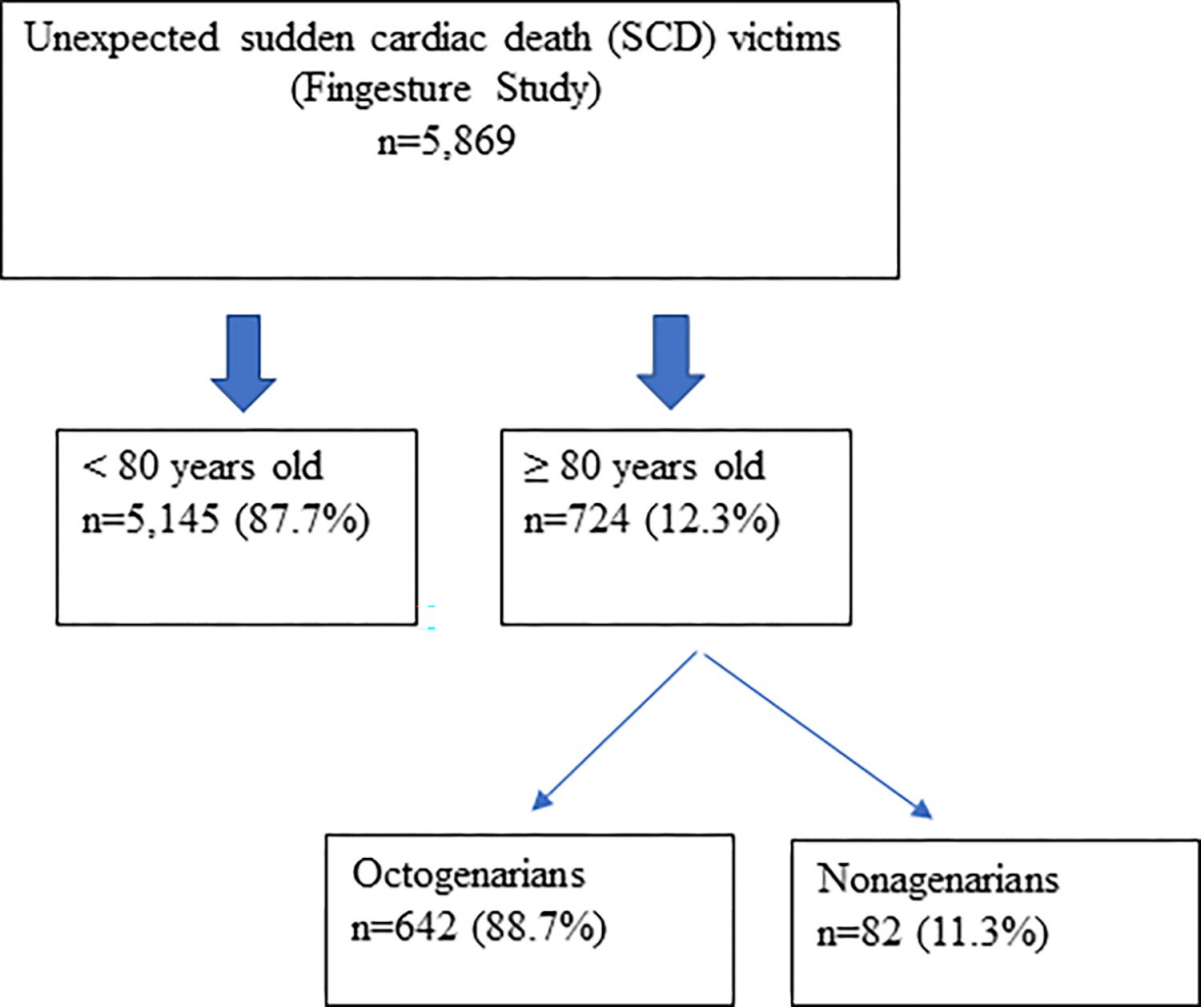
Flow chart of the study population. The data was obtained from the large prospective Fingesture study (Finnish Genetic Study of Arrhythmic Events), which consists of a consecutive series of 5,869 victims of unexpected sudden cardiac death (SCD) in Northern Finland. Medico-legal autopsy is mandatory in cases of unexpected sudden death in Finland. Octogenarians = from 80 to 89 years old. Nonagenarians = from 90 to 99 years old.

Of all SCD victims, 12.3% (724/5,869) were aged ≥ 80 years (Table 1). Of those, 88.7% (642/724) were aged 80–89 years (octogenarians) and the rest (82/724, 11.3%) were aged 90–99 years (nonagenarians) (Table 2, Fig 1). The mean age of the SCD victims ≥ 80 years old was 84.3 years and among those <80 years old, 62.1 years (Table 1). Of the whole study population, 78.9% were males (4,631/5,869). However, the proportion of females in SCD victims increased significantly with age. Among those < 80 years, only 17.7% of the SCD victims were females, whereas among octogenarians 43.0% of the victims were females. Among nonagenarians, the proportion of females was higher than that of males (61.0%) (Tables 1 and 2).

**Table 1:** Characteristics of sudden cardiac death victims.

Characteristic		SCD victims < 80 years (n = 5,145, 87.7%)	SCD victims ≥ 80 years (n = 724, 12.3%)	P-value
Autopsy based data				
Age, years		62.1±10.6	84.3±3.8	< .001
Gender	Male	82.3%	55.0%	< .001
	Female	17.7%	45.0%	
BMI, kg/m^2^		27.9±6.4	25.9±5.3	< .001
Height, cm		171.4±9.2	162±9.2	< .001
Weight, kg		82.4±22.2	68.8±15.7	< .001
Heart weight, g		486.9±130.7	447.5±103.0	< .001
Liver weight, g		1787.3±626.2	1291.5±338.0	< .001
Abdominal fat, cm		2.9±1.5	2.4±1.3	< .001
Ischemic SCD		72.6%	91.0%	< .001
CAD stenosis > 75%		76.2%	82.2%	0.001
Cardiomyopathy		17.5%	4.6%	< .001
Valvular heart disease		3.4%	9.5%	< .001
Hypertensive myocardial disease		5.5%	3.7%	0.051
Myocarditis		1.1%	0.4%	0.08
Degree of myocardial fibrosis	Substantial	11.1%	16.0%	< .001
	Moderate patchy	50.3%	52.5%	
	Scattered mild	30.1%	25.3%	
	None	8.5%	6.2%	
Fatty liver	Worst	18.0%	2.8%	< .001
	Medium	22.3%	10.8%	
	Mild	30.6%	32.2%	
	None	29.1%	54.2%	
Liver cirrhosis	Worst	2.7%	0.3%	< .001
	Medium	6.3%	2.1%	
	Mild	11.2%	7.9%	
	None	79.8%	89.8%	
Place and circumstances of SCD				
During physical activity		15.9%	5.6%	< .001
Sauna		2.6%	5.5%	< .001
Died indoors/outdoors	Indoors	77.9%	84.0%	< .001
	Outdoors	22.1%	16.0%	
Medical history				
CAD		40.9%	62.6%	< .001
Diabetes		20.2%	21.7%	0.40
Heart failure		7.5%	22.2%	< .001
Hypertension		5.5%	3.7%	0.051
Dyslipidemia		13.5%	10.2%	0.02
Acute myocardial infarction		6.8%	10.8%	< .001
Angina pectoris		7.7%	7.0%	0.54
Dyspnea		3.8%	5.0%	0.12
ECG				
LBBB		5.7% (53/936)	6.7% (11/165)	0.72
RBBB		3.6% (34/936)	7.3% (12/165)	0.04
Pathologic Q-waves		14.2% (133/936)	10.9% (18/165)	0.27
WPW		0.2% (2/936)	1.2% (2/165)	0.11

The values are mean ± SD or percentages. Abbreviations: BMI = body mass index, CAD = coronary artery disease, CAD stenosis > 75% = >75% stenosis in one major coronary artery, LBBB = left bundle branch block, RBBB = right bundle branch block, WPW = Wolff-Parkinson-White syndrome. We would like to emphasize that the SCDs were unexpected, and that the medical history data of the victims are not complete. Therefore the medical history data should be interpreted cautiously. ECGs were available from 1,101 of the SCD victims and they were retrospectively collected from the subjects’ previous medical records.

**Table 2:** Characteristics of octogenarian and nonagenarian sudden cardiac death victims.

Characteristic		Octogenarians (n = 642, 88.7%)	Nonagenarians (n = 82, 11.3%)	P-value
Autopsy based data				
Age, years		83.3±2.6	92.1±2.1	< .001
Gender	male	57.0%	39.0%	0.002
	female	43.0%	61.0%	
BMI, kg/m^2^		26.1±5.3	24.1±5.5	0.001
Height, cm		163.3±9.2	159.9±9.2	0.002
Weight, kg		69.7±15.6	61.8±15.1	< .001
Heart weight, g		453.1±103.6	403.6±87.0	< .001
Liver weight, g		1315.0±337.9	1108.7±279.4	< .001
Abdominal fat, cm		2.5±1.3	2.0±1.1	0.003
Ischemic SCD		91.1%	90.2%	0.84
CAD stenosis >75%		81.5%	87.8%	0.20
Cardiomyopathy		4.4%	6.1%	0.57
Valvular heart disease		8.6%	17.1%	0.018
Hypertensive myocardial disease		3.4%	6.1%	0.35
Myocarditis		0.3%	1.2%	0.30
Degree of myocardial fibrosis	Substantial	17.0%	8.5%	0.13
	Moderate patchy	52.6%	51.2%	
	Scattered mild	24.3%	32.9%	
	None	6.2%	7.3%	
Fatty liver	Worst	3.0%	1.2%	0.015
	Medium	11.8%	2.4%	
	Mild	32.7%	28.0%	
	None	52.5%	68.3%	
Liver cirrhosis	Worst	0.3%	0.0%	0.58
	Medium	2.2%	1.2%	
	Mild	8.3%	4.9%	
	None	89.3%	93.9%	
Place and circumstances of death				
During physical activity		6.2%	1.6%	0.24
Sauna		5.9%	2.5%	0.30
Died indoors/outdoors	Indoors	83.5%	87.8%	0.34
	Outdoors	16.5%	12.2%	
Medical history				
CAD		61.3%	72.8%	0.051
Diabetes		21.3%	24.7%	0.56
Heart failure		20.5%	35.9%	0.003
Hypertension		3.4%	6.1%	0.35
Dyslipidemia		10.9%	3.9%	0.07
Acute myocardial infarction		11.5%	5.2%	0.12
Angina pectoris		6.8%	8.9%	0.64
Dyspnea		5.0%	5.2%	1.0
ECG				
LBBB		6.9% (10/144)	4.8% (1/21)	1.0
RBBB		7.6% (11/144)	4.8% (1/21)	0.71
Pathologic Q-waves		12.5% (18/144)	0.0% (0/21)	0.08
WPW		0.7% (1/144)	4.8% (1/21)	0.24

The values are mean ± SD or percentages. Abbreviations: BMI = body mass index, CAD = coronary artery disease, CAD stenosis >75% = >75% stenosis in one major coronary artery, LBBB = left bundle branch block, RBBB = right bundle branch block, WPW = Wolff-Parkinson-White syndrome. We would like to emphasize that the SCDs were unexpected, and that the medical history data of the victims are not complete. Therefore the medical history data should be interpreted cautiously.

### Etiology of sudden cardiac death

**Fig 2:**
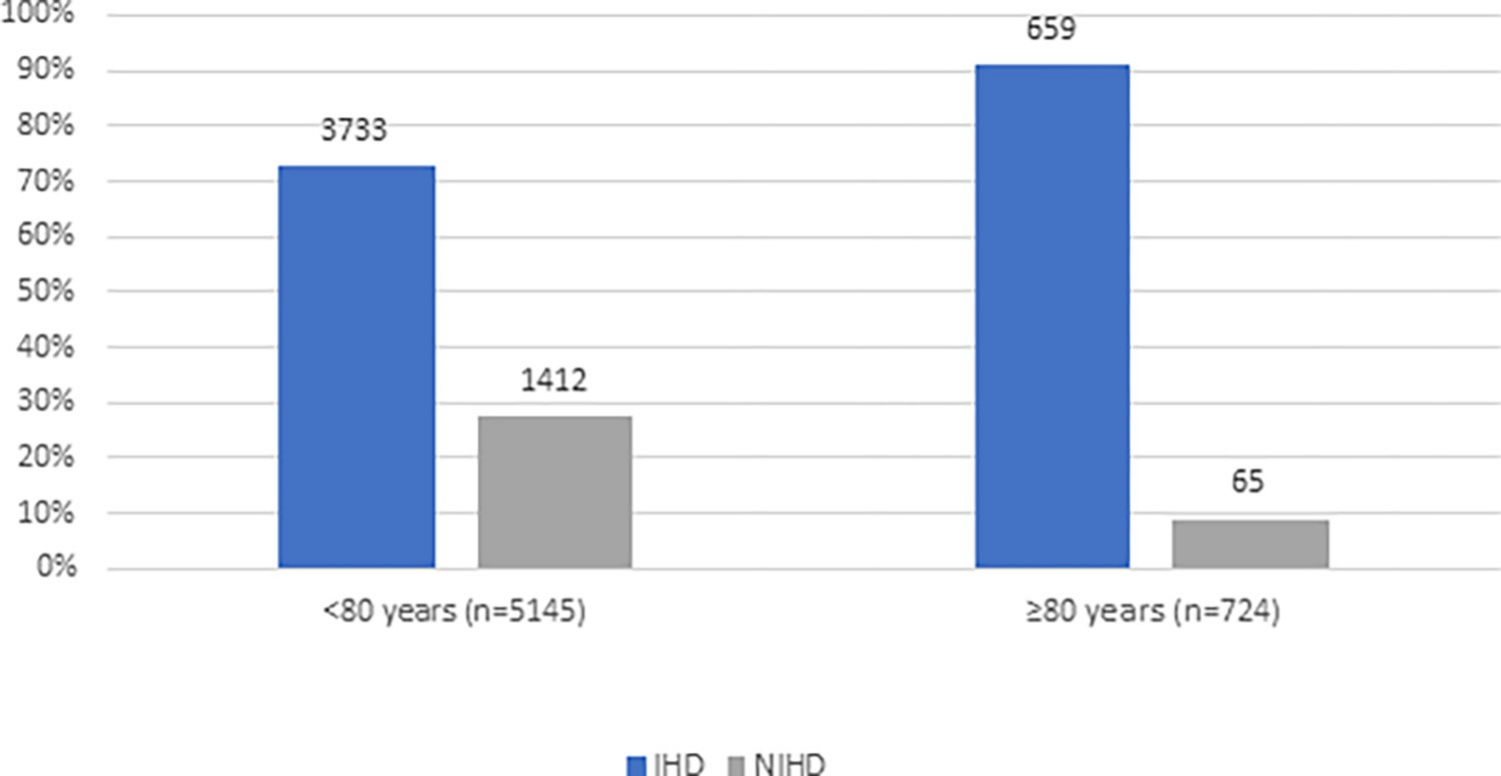
The proportion of ischemic heart disease (IHD) and nonischemic heart disease (NIHD) as a cause of sudden cardiac death (SCD) in <80 years old and ≥80 years old. IHD was more commonly cause of SCD in ≥80 years old than <80 years old (91.0% vs. 72.6%, P < .001). Percentages represent a proportion of cause of SCD in each age group seperately. Values over the bars represent the number of cases in the age group.

Based on autopsy findings, IHD was more common cause of SCD in octo- and nonagenarians than in those under 80 years old. Among those ≥ 80 years, 91.0% of SCDs were due to ischemic cause, whereas among those < 80 years old only 72.6% of SCDs were of ischemic etiology (P < .001) (Table 1, Fig 2). However, there was no statistically significant difference in the proportion of IHD as a cause SCD between octogenarians and nonagenarians (91.1% vs. 90.2%, P = 0.84) (Table 2). IHD was more common among male than among female SCD victims in the group aged 80 years or more (93.0% vs. 88.7%, P = 0.05) (Table 3). More than half (62.6%) of the SCD victims aged ≥ 80 years and less than half (40.9%) of those aged <80 years had a history of CAD prior to SCD (P < .001). Medical history of the SCD victims of the present study is shown in Tables 1–3.

**Table 3:** Characteristics of male and female sudden cardiac death victims aged ≥ 80 years.

Characteristic		Male SCD victims aged ≥ 80 years (n = 398, 55.0%)	Female SCD victims aged ≥ 80 years (n = 326, 45.0%)	P-value
Autopsy based data				
Age, years		83.7±3.3	85.1±4.2	< .001
BMI, kg/m^2^		25.3±4.9	26.6±5.8	0.002
Height, cm		168.5±7.1	156.0±6.4	< .001
Weight, kg		72.1±15.4	64.9±15.3	< .001
Heart weight, g		480.1±103.9	407.8±86.8	< .001
Liver weight, g		1331.9±331.7	1242.0±339.5	< .001
Abdominal fat, cm		2.2±1.2	2.7±1.4	< .001
Ischemic SCD		93.0%	88.7%	0.05
CAD stenosis >75%		83.5%	80.6%	0.36
Cardiomyopathy		3.3%	6.1%	0.07
Valvular heart disease		8.0%	11.3%	0.16
Hypertensive myocardial disease		3.5%	4.0%	0.84
Myocarditis		0.5%	0.3%	1.00
Degree of myocardial fibrosis	Substantial	20.4%	10.7%	0.005
	Moderate patchy	50.0%	55.5%	
	Scattered mild	24.4%	26.4%	
	None	5.3%	7.4%	
Fatty liver	Worst	3.3%	2.1%	0.85
	Medium	10.6%	11.0%	
	Mild	32.2%	32.2%	
	None	54.0%	54.6%	
Liver cirrhosis	Worst	0.0%	0.6%	0.39
	Medium	2.3%	1.8%	
	Mild	8.5%	7.1%	
	none	89.2%	90.5%	
Place and circumstances of SCD				
During physical activity		8.3%	2.5%	0.0060
Died at sauna		6.2%	4.8%	0.50
Died indoors/outdoors	Indoors	78.9%	90.2%	< .001
	Outdoors	21.1%	9.8%	
Medical history				
CAD		61.5%	63.9%	0.54
Diabetes		22.2%	21.0%	0.78
Heart failure		19.7%	25.3%	0.08
Hypertension		3.5%	4.0%	0.84
Dyslipidemia		11.1%	9.0%	0.38
Acute myocardial infarction		12.9%	8.3%	0.07
Angina pectoris		7.0%	7.1%	1.0
Dyspnea		5.4%	4.5%	0.61
ECG				
LBBB		7.3% (6/82)	6.0% (5/83)	0.77
RBBB		12.2% (10/82)	2.4% (2/83)	0.02
WPW		1.2% (1/82)	1.2% (1/83)	1.0
Pathologic Q-waves		14.6% (12/82)	7.2% (6/83)	0.14

The values are mean ± SD or percentages. Abbreviations: BMI = body mass index, CAD = coronary artery disease, CAD stenosis >75% = >75% stenosis in one major coronary artery, LBBB = left bundle branch block, RBBB = right bundle branch block, WPW = Wolff-Parkinson-White syndrome. We would like to emphasize that the SCDs were unexpected, and that the medical history data of the victims are not complete. Therefore the medical history data should be interpreted cautiously.

Based on autopsy findings, NIHD was a less common etiology of SCDs among those ≥ 80 years than among those < 80 years (Table 1). The proportion of cardiomyopathies as a cause of SCD did not differ statistically significantly between octogenarians and nonagenarians (4.4% vs. 6.1%, P = 0.57) (Table 2) or between males and females among those ≥ 80 years (3.3% vs. 6.1%, P = 0.07) (Table 3). On autopsy, valvular heart disease was a more common finding in SCD victims aged ≥ 80 years than in those aged < 80 years (9.5% vs. 3.4%, P < .001) (Table 1) and in nonagenarian SCD victims than in octogenarian SCD victims (17.1% vs. 8.6%, P = 0.02) (Table 2). The proportion of valvular heart disease did not differ significantly between males and females in SCD victims aged ≥ 80 years (8.0% vs. 11.3%, P = 0.16) (Table 3). The differences in proportions of hypertensive myocardial disease or myocarditis as autopsy findings did not reach statistical significance between SCD victims ≥ 80 years and those < 80 years (3.7% vs. 5.5%, P = 0.051, 0.4% vs. 1.1%, P = 0.08, respectively) (Table 1).

### Autopsy findings/characteristics

Four-fifths (82.2%) of the ischemic SCD victims ≥ 80 years had ≥ 75% stenosis in one major coronary artery measured in the cross-sectional area, whereas 76.2% of the ischemic SCD victims < 80 years had ≥ 75% stenosis (P = 0.001) (Table 1).

**Fig 3:**
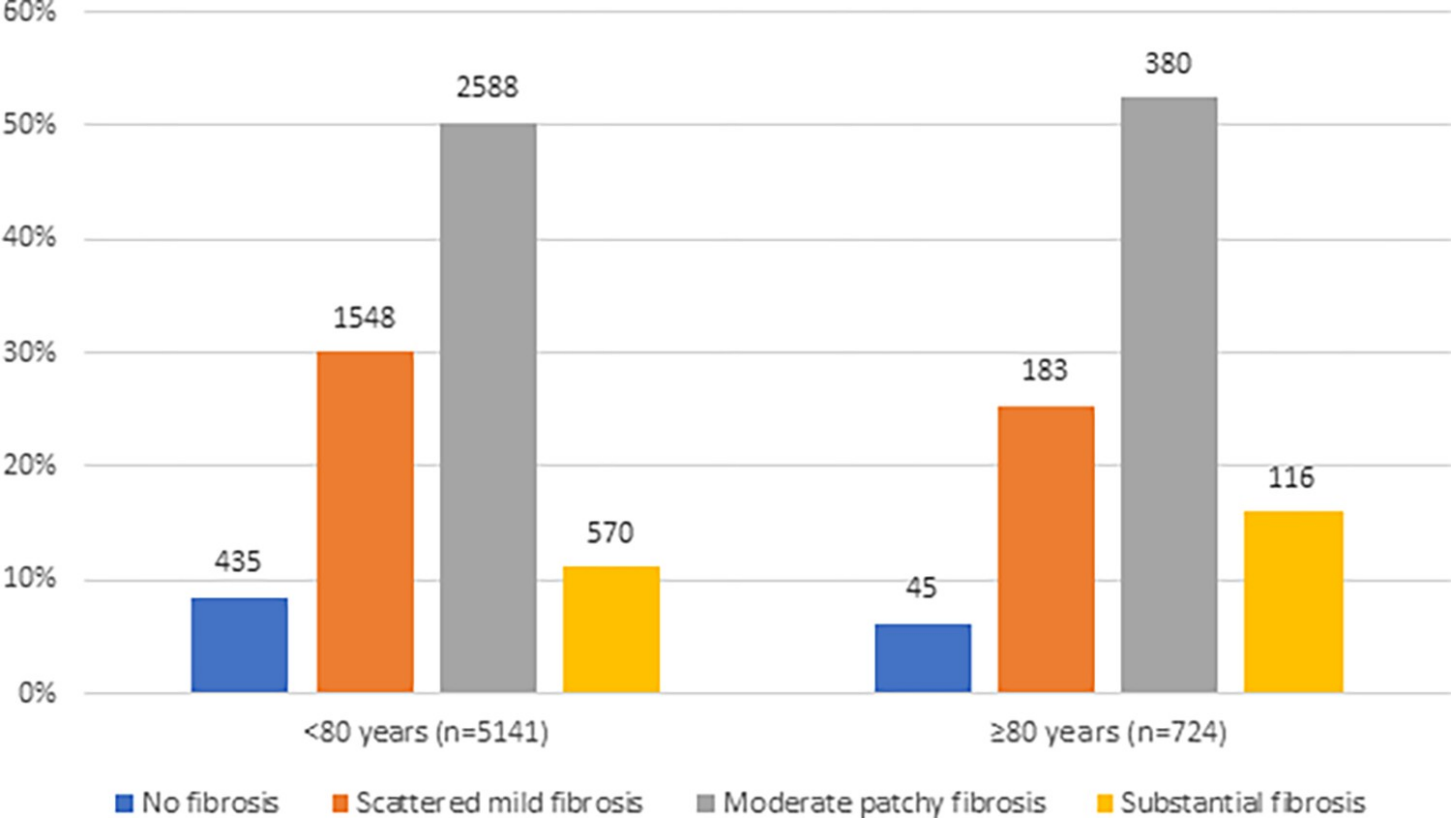
Degree of myocardial fibrosis in <80 years old and ≥80 years old sudden cardiac death (SCD) victims. SCD victims ≥80 years old had more commonly worse stage of fibrosis than <80 years old (P < .001). Percentages represent a proportion of cases in each age group seperately. Values over the bars represent the number of cases in the age group.

Body mass index (BMI) (P < .001), abdominal fat thickness (P < .001), heart weight (P < .001) and liver weight (P < .001) were lower among SCD victims aged ≥ 80 years than among victims aged < 80 years. However, even in victims ≥ 80 years, the average heart weight was higher than the criteria used for cardiomegaly in the study by Roberts and Shirani (> 350 g in females, > 400 g in males) (7) (Tables 1 and 3). The proportion of females was higher and the body weight was lower in SCD victims aged ≥ 80 years than victims aged < 80 years. (Table 1). Heart weight, liver weight, and abdominal fat thickness had significant correlations with the body weight (p<0.001, the Pearson correlation coefficients: 0.619, 0.641, 0.688, respectively). All of these characteristics were also lower among nonagenarians than among octogenarians, as was the frequency of the occurrence of fatty liver (P = 0.02) (Table 2). In addition, having fatty liver (P < .001) or liver cirrhosis (P < .001) was significantly less frequent among SCD victims aged ≥ 80 years than among those aged < 80 years. On the contrary, having severe fibrosis in myocardium (P < .001) was more frequent in those ≥80 years than in those < 80 years (Table 1, Fig 3). Female SCD victims aged 80 years or more had higher BMI (P < .001) and more abdominal fat (P < .001) than male ones in the same age group, whereas male’s heart (P < .001) and liver weight (P < .001) was greater than that of females. Also, males ≥ 80 years had more frequently worse stage of fibrosis in myocardium (P = 0.005) than females ≥ 80 years (Table 3).

### Place and circumstances of sudden cardiac death

SCD victims 80 years or older were less likely to die during physical activity than those under 80 years old (5.6% vs. 15.9%, P < .001). Dying in sauna was more common among those ≥ 80 years than among those <80 years (5.5% vs. 2.6%, P < .001) (Table 1). Males ≥ 80 years old died more commonly during physical activity than females of the same age group (8.3% vs. 2.5%, P = 0.006) (Table 3). The probability of experiencing SCD in sauna did not differ significantly between octogenarians and nonagenarians or between males and females ≥ 80 years (Tables 2 and 3).

Most of the SCDs in the whole study population occurred indoors (78.7%, 4,609/5,860). SCD occurring outdoors or SCD occurring in a person who had recently been outdoors was more likely to happen among those aged < 80 years than among those aged ≥ 80 years (22.1% vs. 16.0%, P < .001) (Table 1). Also, among SCD victims ≥ 80 years, males were more likely to experience SCD outdoors or after having recently been outdoors than females (21.1% vs. 9.8%, P < .001) (Table 3).

## Discussion

Based on findings of medico-legal autopsies in a large consecutive series of unexpected SCD victims, IHD was a more common etiology of SCD among subjects aged ≥ 80 years than among those aged < 80 years. Consequently, NIHD was a less common etiology of SCDs among those ≥ 80 years than among those <80 years. The proportion of IHD and the proportion of NIHD as a cause of SCD between octogenarians and nonagenarians was similar. The proportion of females in SCD victims increased with age. The SCD victims aged ≥ 80 years had more extensive CAD and more severe fibrosis in myocardium than those aged < 80 years. The SCD victims ≥80 years were leaner, had lower heart and liver weight, smaller abdominal fat thickness, and had less frequently fatty liver or liver cirrhosis. Heart weight, liver weight, and abdominal fat thickness had significant direct correlations with the body weight, which may largely explain their aforementioned differences between SCD victims ≥80 years and <80 years as body weight was lower in the victims ≥80 years. SCD victims 80 years old or older died more commonly in sauna than those < 80 years. SCD occurred less commonly outdoors or when having recently been outdoors in those ≥ 80 years than in those < 80 years.

IHD is a major cause of mortality and SCD in middle-aged and elderly subjects, and its prevalence increases with age [7,8]. Our present findings are in alignment with this concept, showing that the proportion of ischemic etiology of SCD was more dominant in victims aged 80 years or more than in younger ones. Myocardial hypertrophy and fibrosis were very prevalent findings in the SCD victims of the present study, and they may have contributed to the occurrence of SCD both in IHD ja NIHD. Recent findings have shown that acute plaque complications may be less common initiators of SCD in CAD than previously thought suggesting the importance of arrhythmic substrate induced ventricular tachyarrhythmias as a cause for SCD in CAD patients [9]. Autopsy-based studies have also found that CAD in the elderly often has characteristics of extensive disease, such as obstructive CAD, multivessel disease and calcifications [7,10]. Our study extends these observations to SCD victims by showing that victims aged ≥ 80 years had more extensive CAD, having more frequently more than 75% stenosis in major coronary arteries measured in the cross-sectional area. According to an autopsy study by Roberts and Shirani including 490 elderly cases, in 228 of which the cause of death was cardiac, calcific deposits in coronary arteries were more common in nonagenarians than octogenarians, however, the narrowing of the coronary arteries was similar [10]. In our present study including only SCD victims, the proportion of ischemic etiology of SCD and the autopsy-based severity of CAD were similar between octogenarians and nonagenarians. It is noteworthy that only a very small proportion of SCDs was due to cardiomyopathy in subjects aged 80 years or more. The prevalence of valvular heart disease in SCD victims increased with age.

SCD is known to be more common in males than in females, particularly in middle-aged subjects. However, the difference decreases with age [11,12]. Previous studies have reported that death rates for SCD do not differ between genders after the age of 85 years [11,13], although some studies claim that elderly males have a higher rate for SCD than elderly females [14]. In the present study, the proportion of females among SCD victims increased with age, resulting in higher proportion of females than males among nonagenarians. The larger proportion of females in nonagenarians in our study could be explained by the larger proportion of females in older age groups as females have higher life expectancy [15].

In the present study, the SCD victims aged ≥ 80 years had more often severe fibrosis in myocardium than those < 80 years. Fibrosis and scars in myocardium may indicate previous and healed myocardial infarction and make the heart vulnerable to life-threatening ventricular tachyarrhythmias. In the aforementioned autopsy study in the elderly including both cardiac and non-cardiac cases, 47% of the subjects had grossly visible evidence of left ventricular necrosis or fibrosis, with a similar proportion in octogenarians and nonagenarians [10]. In our study including only SCD victims, the percentage of cases with myocardial fibrosis on meticulous examinations was similar in octogenarians and nonagenarian, of which a vast majority had at least some degree of fibrosis. In the present study, the average heart weight was higher than the criteria used for cardiomegaly in the study by Roberts and Shirani [10] (> 350 g in females, > 400 g in males) in both old males and females, but lower than the heart weights of those aged < 80 years. The latter finding is further underlined by the observation that the nonagenarians had lower heart weight than octogenarians. Similar heart weights as in the present study were found in the old in the autopsy study by Roberts et al., and in accordance with our observations, the heart weight was lower in nonagenarians than in octogenarians [10]. In addition to lower heart weight, we found that the old had lower liver weight, were leaner and had less fatty liver, which is most likely due to unintentional weight loss associated with aging [16].

Strenuous physical activity seems to increase the risk of SCD temporarily and the least fit population is at the highest risk [17]. However, in our study, the old experienced less frequently SCD during or after physical activity, which is in line with the other studies [18,19]. The trend is understandable since physical activity decreases with age [20,21], especially moderate and strenuous exercise [21]. Previous studies present that even though the incidence of sport-related SCD declines after middle age in the whole population, the incidence increases with age in those who exercise regularly [18,22].

Many studies have reported that males experience more commonly SCD during physical activity than females [18,19]. We noticed the same trend in the old in the present study. It might not be fully explained with men being more physically active than females, since many studies have reported that there is no significant difference in the daily activity between elderly males and females [20,21]. Previous data has also suggested that the amount of moderate and vigorous physical activity does not differ substantially between males and females among the elderly [21]. However, the proportion of females is higher among the older age groups and the amount of physical activity declines with age [20]. Interestingly, old males had more ischemic SCD and substantial myocardial fibrosis representing scars than old females, both of which have been associated with SCD during physical activity [17].

Even though sauna bathing is associated with many health benefits [23], and Laukkanen T et al. found a reverse link between the duration and frequency of sauna bathing and SCD [24], some studies have reported that sauna bathing may be associated with increased risk of SCD [23]. In most of those cases, alcohol has been evaluated to play a large role [23]. In our study, the SCD victims aged ≥ 80 years had more commonly SCD associated with sauna bathing than those aged < 80 years. The old seem to be more susceptible to the negative effects of sauna bathing, such as dehydration, hypotension and cardiac arrhythmias [23], which can trigger SCD.

Recently, it has been paid attention that the subgroups of subjects, such as the elderly and women, have been underrepresented in the randomized clinical trials. Our present study included all female and male unexpected SCD victims from all age groups from the certain area. We found that the proportion of females among unexpected SCD victims increased during aging. In the vast majority of the old SCD victims, regardless of gender, the main etiologic factor was IHD. One of the main aims of modern medicine is to decrease mortality and prolong lives. In the developed countries, substantial proportion of the subjects aged ≥ 80 years are active and have good quality of life. Our observations emphasize the importance of effective treatments of subclinical and clinical IHD and their risk factors for preventing SCD in the old people.

The present study has some limitations. The classification of the causes of SCD to IHD and NIHD was based mainly on autopsy findings, which may have caused potential bias in some individual cases. However, the classification was based on meticulous macroscopic and microscopic examinations of autopsy findings done by a very experienced forensic pathologist. As stated in the methods, the cause of SCD was classified as IHD or NIHD according to specific criteria. The data was collected from Northern Finland, where population is largely ethnically homogenous. This may have increased the consistency of the present findings. However, the findings may not be generalized in other populations. Low frequency of some pathological findings, such as myocarditis, does not allow a definite conclusion about the difference in their prevalence between the SCD victims ≥ 80 years and < 80 years.

In conclusion, our present findings in a large consecutive series of SCD victims confirm the significance of IHD and reveal its magnitude as a cause of unexpected SCD in subjects ≥ 80 years. Our study also provides information on the characteristics of unexpected SCD in SCD victims aged ≥ 80 years.
